# Supplementary material for: Accelerated discovery of perovskite solid solutions through automated materials synthesis and characterization
Source: Nat Commun. 2024 Aug 2;15:6554. doi: 10.1038/s41467-024-50884-y (PMC11297172; doi:10.1038/s41467-024-50884-y)
Supplement: Supplementary file 1 — Supplementary Information [file 41467_2024_50884_MOESM1_ESM.pdf]

Supplementary Information

Accelerated Discovery of Perovskite Solid Solutions through Automated Materials Synthesis and Characterization

Mojan Omidvar[1], Hangfeng Zhang[1], Achintha Avin Ihalage[1], Theo Graves Saunders[1], Henry Giddens[1], Michael Forrester[2], Sajad Haq[2], Yang Hao[1]*

[1] School of Electronic Engineering and Computer Science, Queen Mary University of London, Mile End Rd, Bethnal Green, E1 4NS, United Kingdom

[2] QinetiQ, Cody Technology Park, Farnborough, Hampshire, GU14 0LX, United Kingdom

E-mail*: y.hao@qmul.ac.uk.

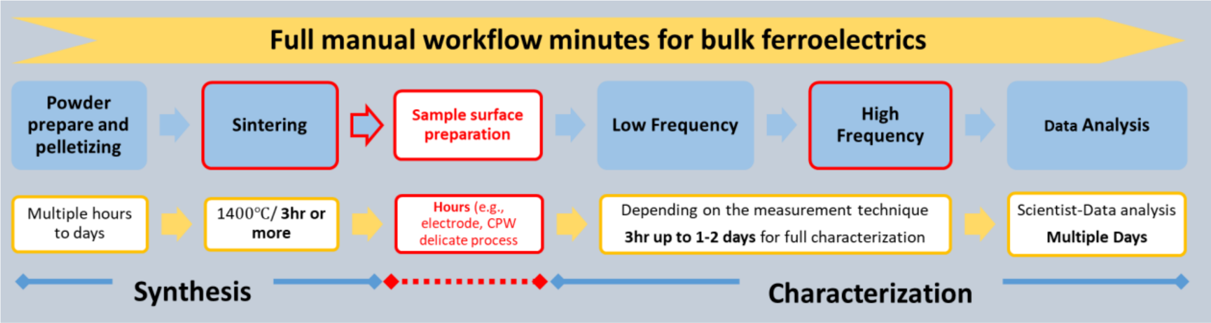

Supplementary Figure 1. Manual synthesis and characterisation timeline. The average time required for the manual workflow of synthesis and characterization of bulk ferroelectrics such as BST. Red outlines indicate the steps selected for automation.

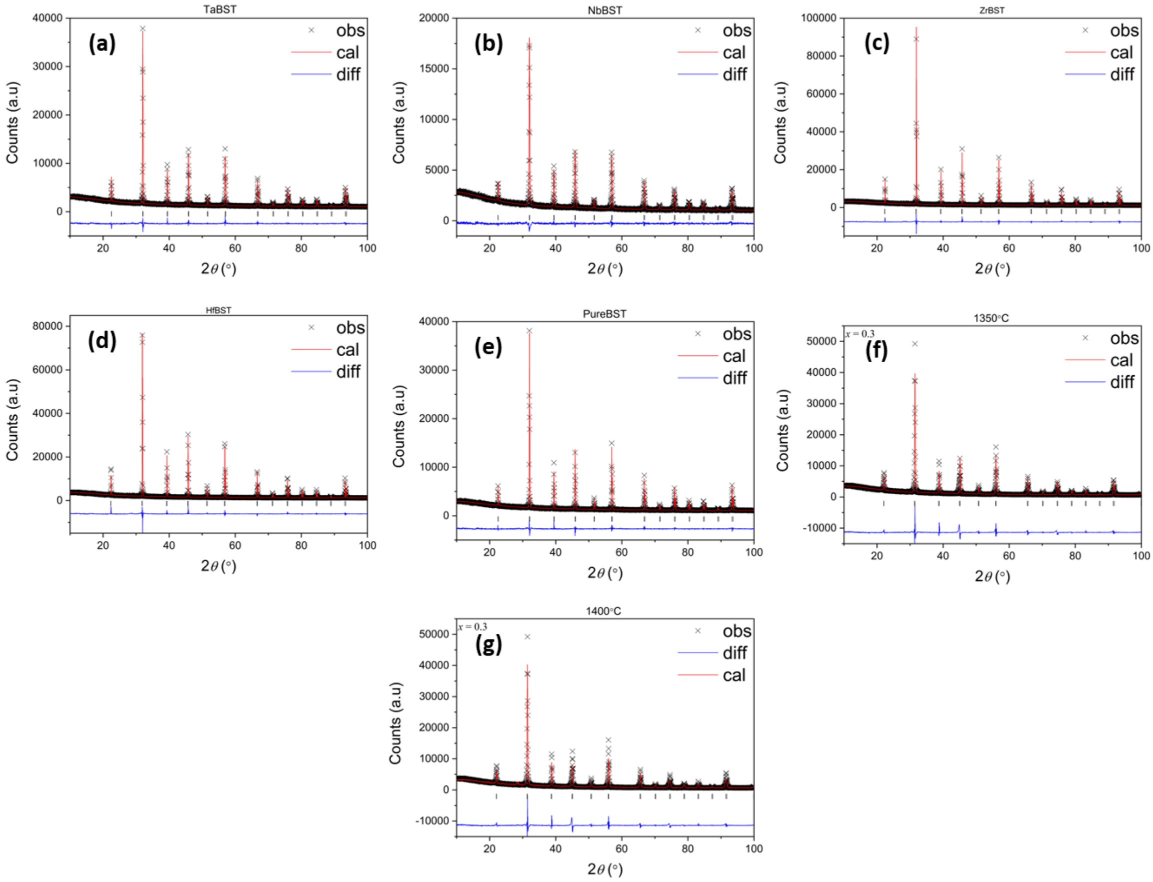

Supplementary Figure 2. XRD patterns. Rietveld fitted XRD patterns of: (a) to (e) B site (Ta/Nb/Zr/Pure/Hf) doping in BST rapidly sintered using the high-throughput setup, (f) BTS12-1350°C, and (g) BTS12-1400°C.

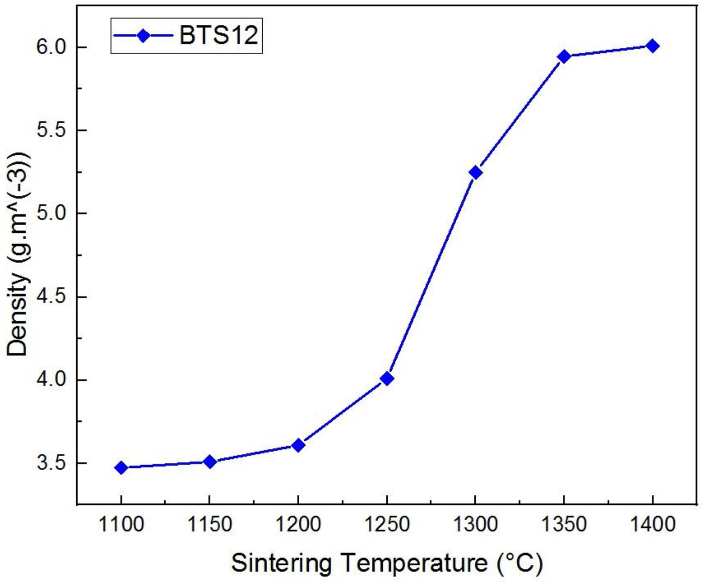

Supplementary Figure 3. Sintering temperature vs. density. Density of BTS12 samples sintered at different temperatures.

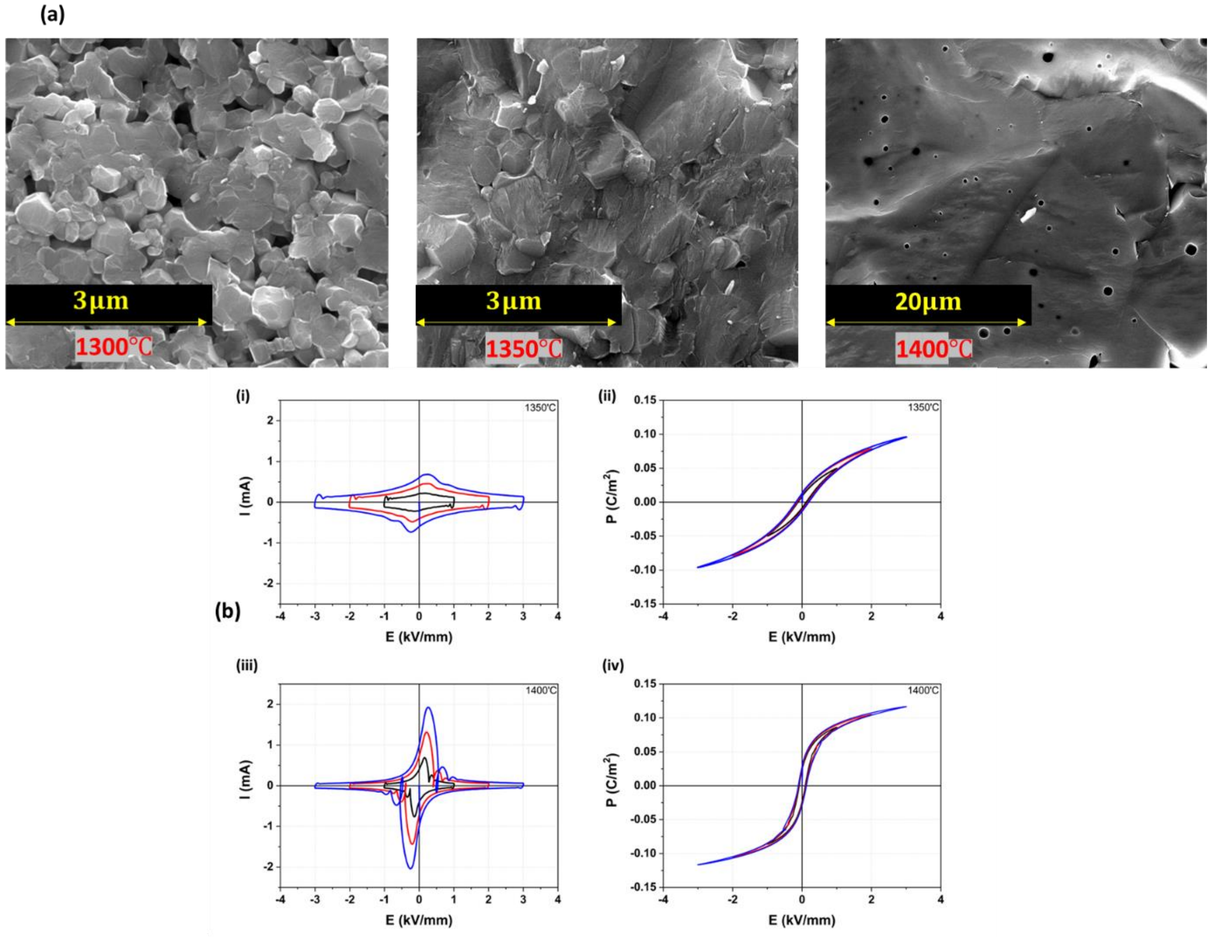

Supplementary Figure 4. **Impact of sintering on BTS12.** a) The SEM images of BTS12-1300°C, BTS12-1350°C, BTS12-1400°C, (b) P-E/I-E loops of BTS12-1350°C and BTS12-1400°C measured at 10 Hz at room temperature.

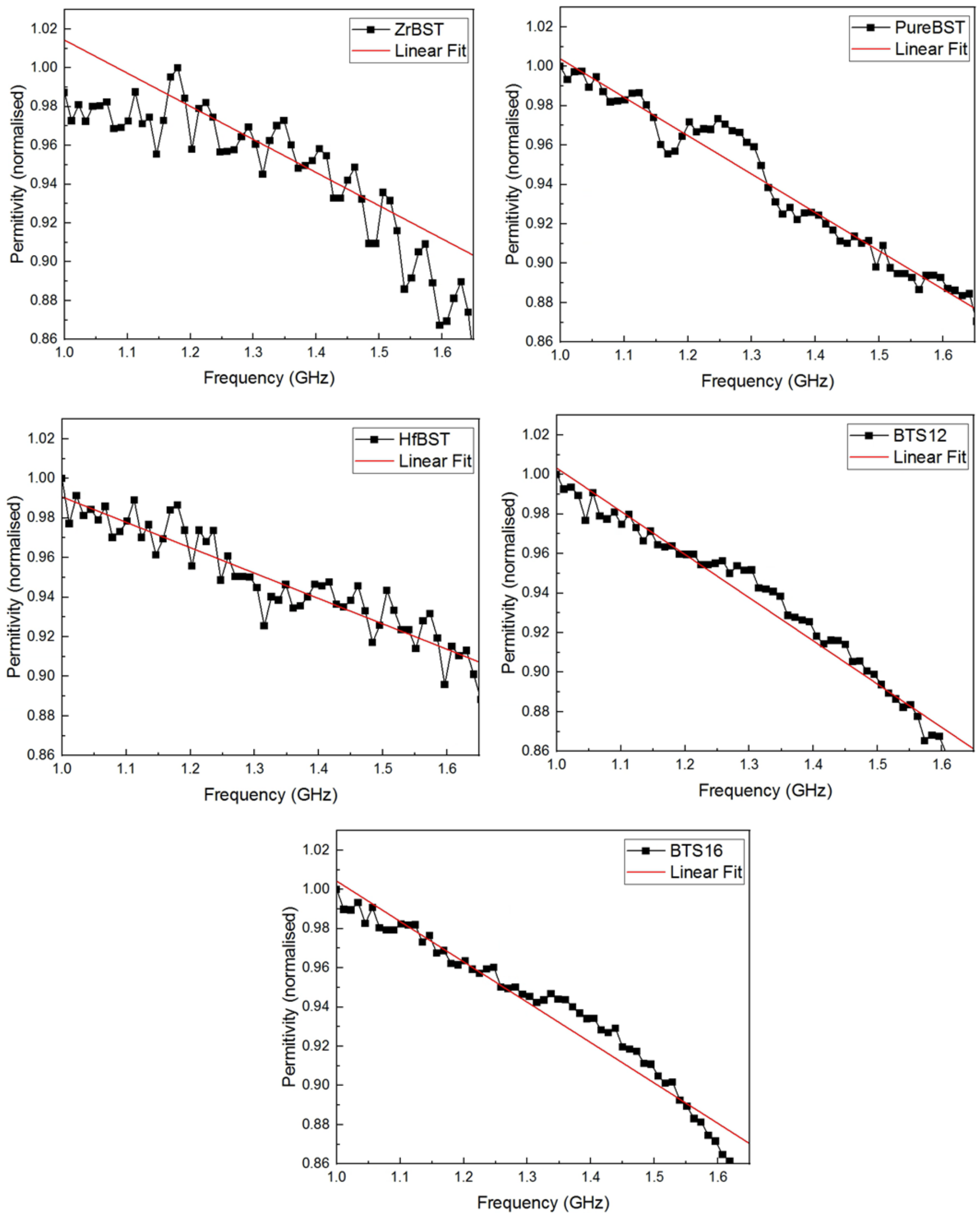

Supplementary Figure 5. Dielectric permittivity vs. frequency. Frequency dependence of normalized dielectric permittivity measured at room temperature with linear fitting for selected samples using CPW technique.

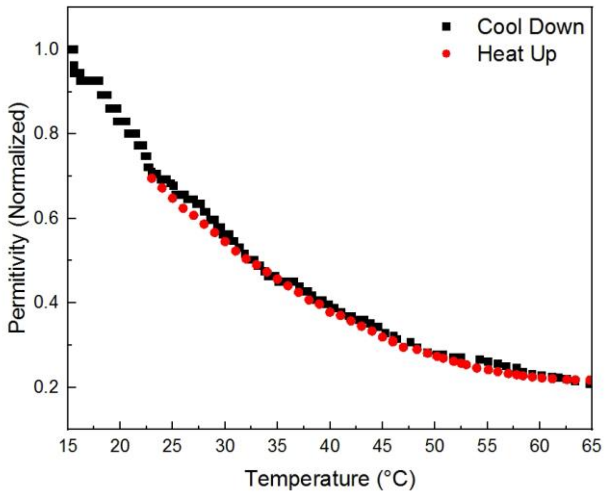

Supplementary Figure 6. Temperature dependence of normalized dielectric permittivity. Measurements during heating and cooling samples using automated sensor.

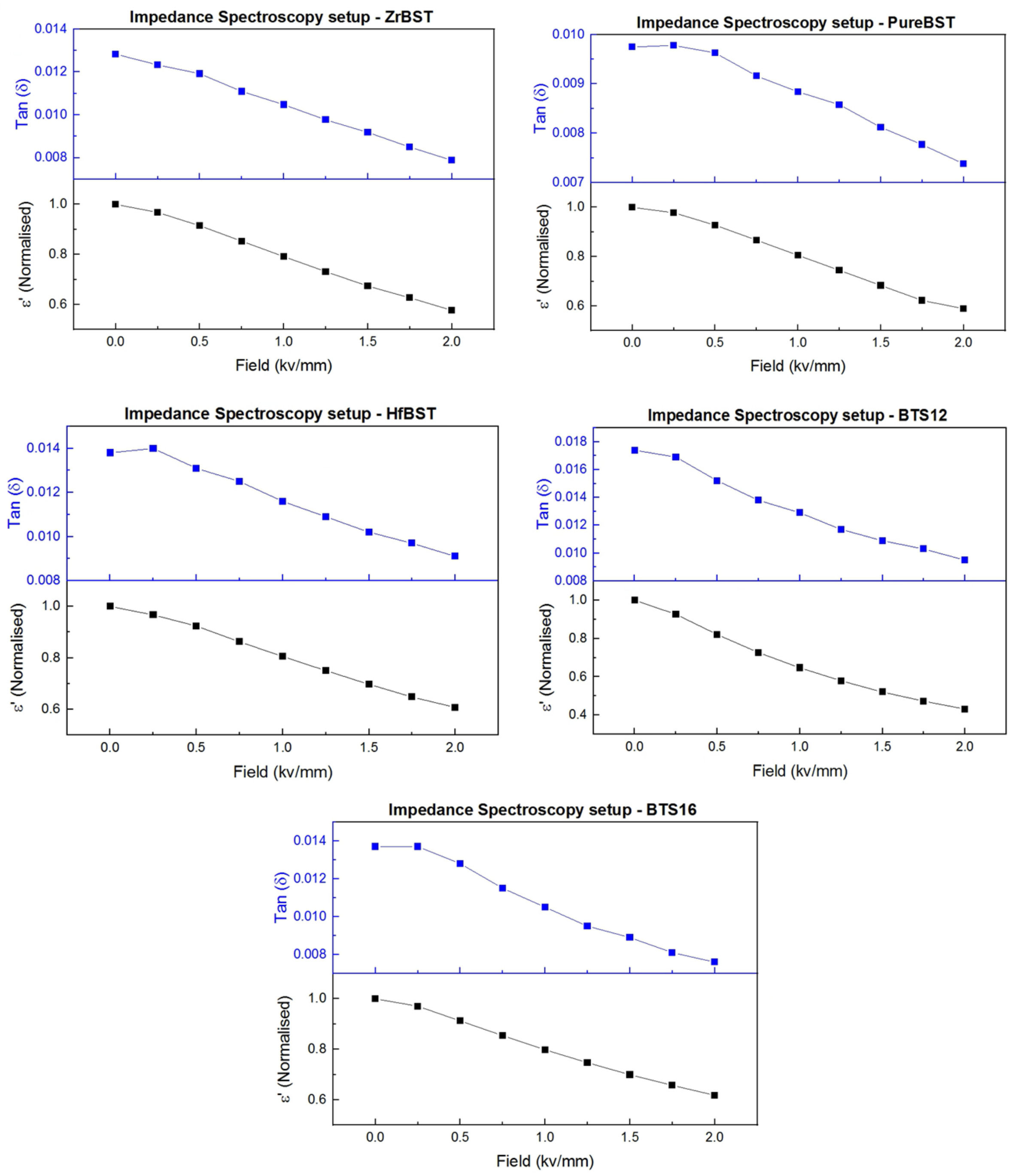

Supplementary Figure 7. Electric field dependence of dielectric loss and permittivity. Electric field dependence of dielectric loss (blue) and permittivity (black) measured at 100kHz for selected samples.

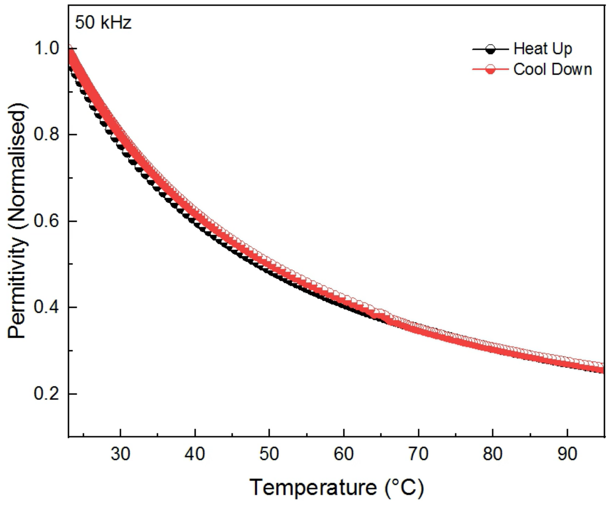

Supplementary Figure 8. Heating/Cooling effects on permittivity. Temperature dependence of normalized dielectric permittivity measured at 50 kHz on heating and cooling.

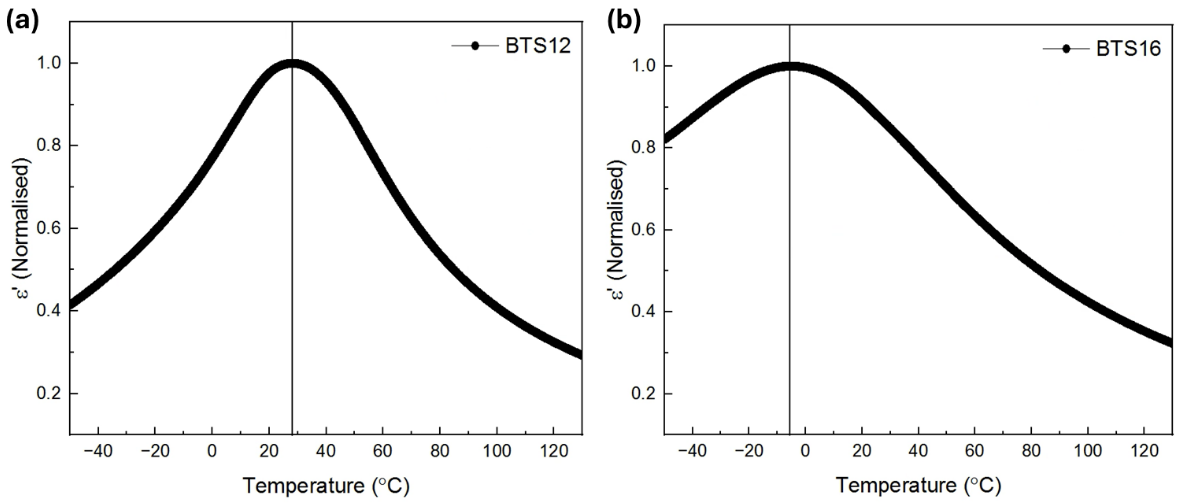

Supplementary Figure 9. Curie temperature measurements. Curie temperature measurement for (a) BTS12 and (b) BTS16.

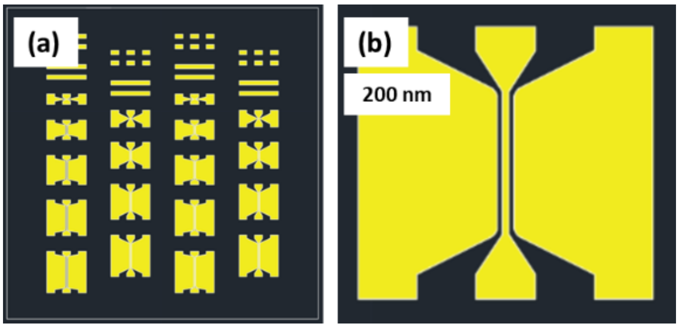

Supplementary Figure 10. CPW patterns. Top-down images of CPW patterns used for measuring the dielectric permittivity and tunability of all five selected samples. (a) The mask sputtered on all samples. (b) Straight CPW transmission line with transition used.

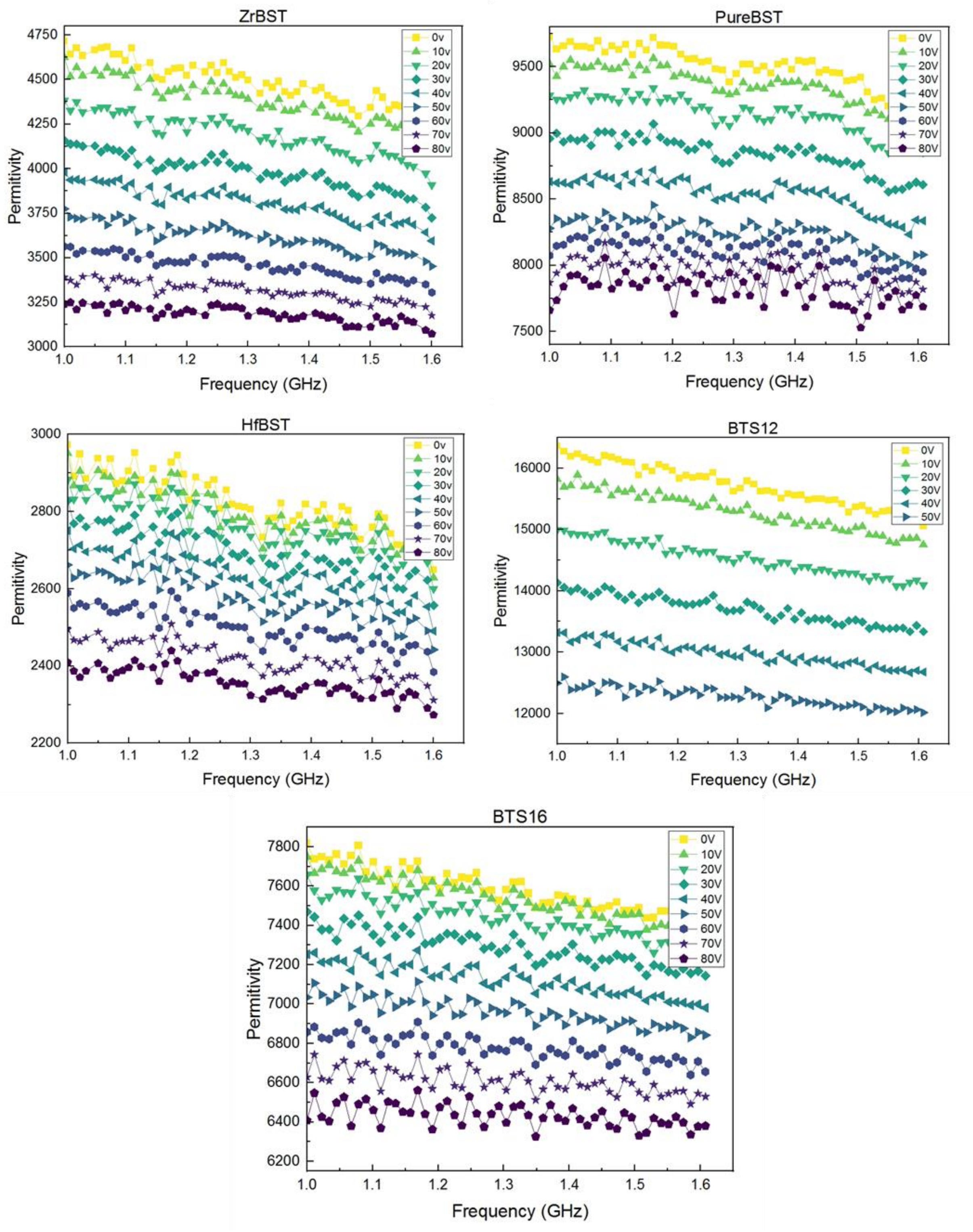

Supplementary Figure 11. Microwave permittivity under DC bias. Dielectric permittivity of the five selected samples measured at microwave frequencies under DC bias field.

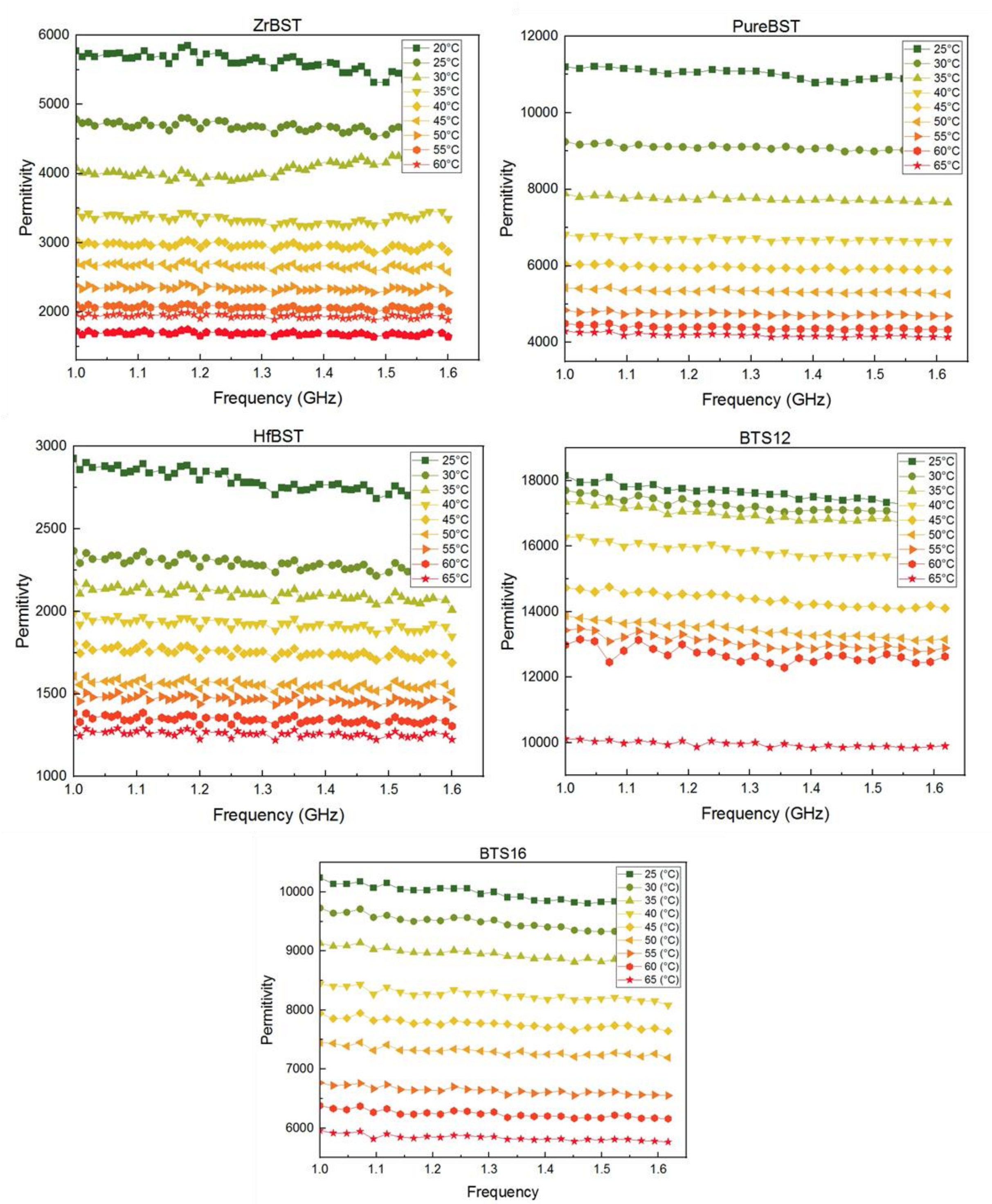

Supplementary Figure 12. Microwave permittivity under temperature bias. Dielectric permittivity of the five selected samples measured at microwave frequencies under different temperatures.

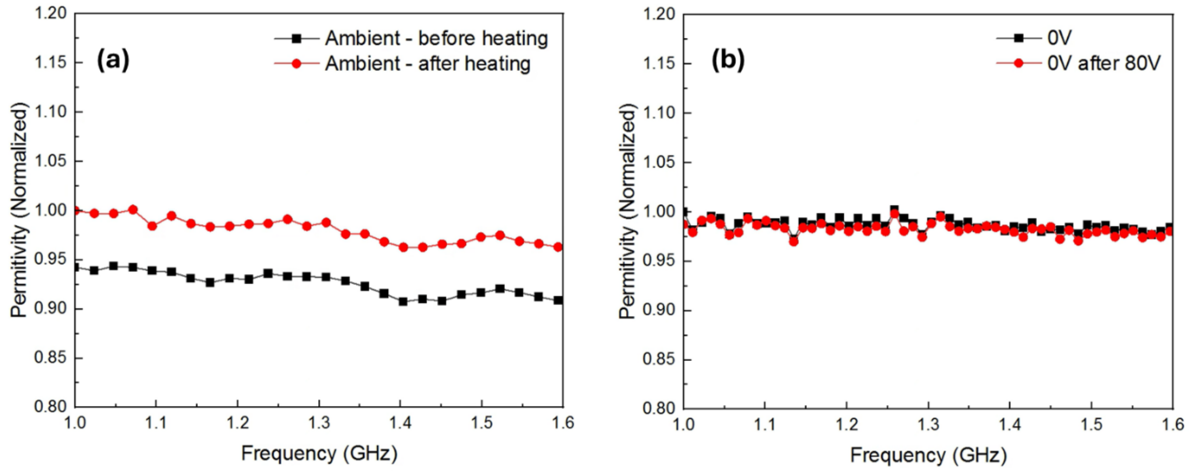

Supplementary Figure 13. Permittivity measurement error with CPW. Microprobe SD system Error measurements. Frequence dependence of normalized dielectric permittivity at room temperature before and after (a) heating process and (b) bias DC field.

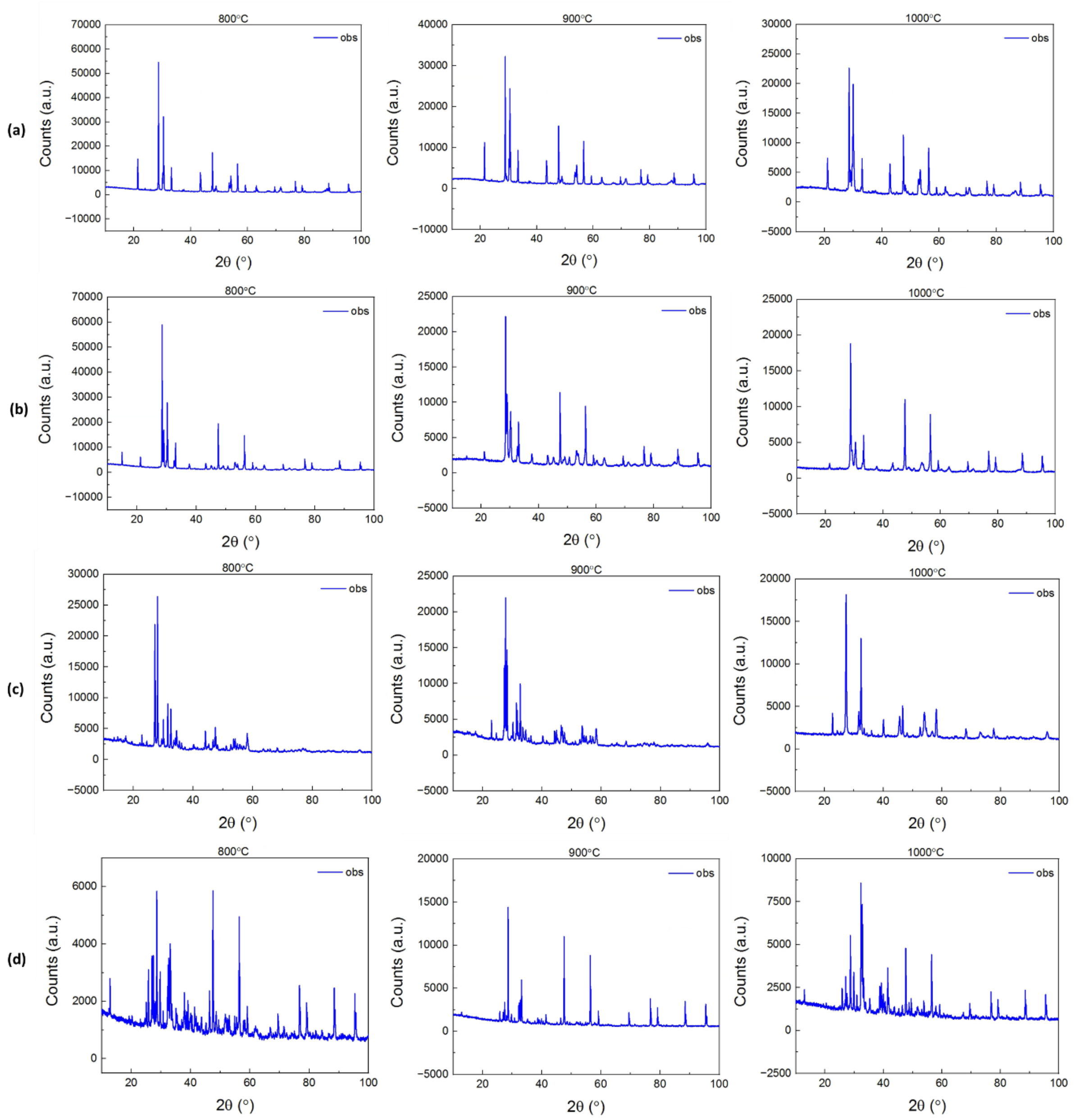

Supplementary Figure 14. XRD patterns of ML-predicted compositions. XRD patterns of ML-predicted compositions calcining at different temperatures; namely, (a) $(Sr_{0.5}Pb_{0.5})CeO_3$, (b) $(Sr_{0.2}Pb_{0.8})CeO_3$, (c) $(La_{0.3}Bi_{0.7})CrO_3$ and (d) $(K_{0.5}Bi_{0.5})CeO_3$.
