# Supplementary material for: Accelerated discovery of perovskite solid solutions through automated materials synthesis and characterization
Source: Nat Commun. 2024 Aug 2;15:6554. doi: 10.1038/s41467-024-50884-y (PMC11297172; doi:10.1038/s41467-024-50884-y)
Supplement: Supplementary file 3 — Description of Additional Supplementary Files [file 41467_2024_50884_MOESM3_ESM.pdf]

Description of Additional Supplementary Files

File Name: Supplementary Movie 1

Description: This video demonstrates the automated system in operation, performing automated pellet fabrication through solid-state sintering, temperature tuning, and conducting real-time measurements and analysis of dielectric properties.
